# Management of Chondroblastoma in Pediatric Patients: 21 Years of Single-Center Experience

**DOI:** 10.3390/children11060672

**Published:** 2024-05-31

**Authors:** Hakan Koray Tosyalı, Hüseyin Kaya, Burcin Kececi, Dündar Sabah

**Affiliations:** 1 Department of Orthopedics and Traumatology, Faculty of Medicine, Celal Bayar University, 45140 Manisa, Türkiye; 2 Department of Orthopedics and Traumatology, Faculty of Medicine, Ege University, 35040 Izmir, Türkiye; huseyin.kaya@ege.edu.tr (H.K.); burcin.kececi@ege.edu.tr (B.K.); dundar.sabah@ege.edu.tr (D.S.)

**Keywords:** chondroblastoma, epiphysis, children

## Abstract

Background: Chondroblastoma (CB), a rare benign bone tumor that produces chondrocytes, often develops in the epiphysis or apophysis of children and young adults. The treatment of these rare tumors is complex. The standard treatment protocol involves curettage with local adjuvants and bone graft or cement application. The authors examined 38 CBs to determine risk factors for local recurrence, complications, and functional outcomes following epiphyseal curettage. Methods: Twenty-two girls and sixteen boys aged 10 to 17 years with histologically confirmed chondroblastoma who arrived at our hospital between January 2000 and June 2021 were reviewed retrospectively. Clinical data, radiographic images, histological results, treatment, functional outcomes, and the local recurrence rate were examined—surgical treatment involved total tumor curettage, followed by bone grafting and adjuvant techniques. Local recurrences have also been reported. Results: The most frequently affected site was the proximal femur. Sites of involvement included the proximal femur in 10 (26.3%) cases, the proximal tibia in 8 (20.8%), the humerus in 5 cases (13.2%), the distal tibia in 4 cases (10.5%), the distal femur in 3 cases (7.9%), the supracetabular region in 3 cases (7.9%), the talus in 1 case (2.6%), the calcaneus in 1 case (2.6%), the scapula in 1 case (2.6%), the lumbar spine in 1 case (2.6%), and the iliac bone in 1 (2.6%) patient. The mean follow-up was 144.2 months (24 to 276). The local recurrence rate was 7.9%. The mean Musculoskeletal Tumor Society (MSTS) score was 28.3 points (17 to 30). The mean duration of symptoms at presentation was 5.8 (range, 1 to 28) months. Conclusion: Aggressive curettage and bone grafting resulted in local control and good outcomes in most pediatric patients. In a relatively small proportion of cases, long-term complications and recurrence can occur due to growth plate damage and late diagnosis. In patients admitted to the pediatric clinic with pain, which is often accompanied by localized edema and joint effusion, early detection via advanced radiological scans (X-ray, CT, or MRI) may prevent delays in diagnosis.

## 1. Introduction

Chondroblastoma (CB) is a rare, non-cancerous bone tumor that is locally aggressive and rarely spreads to other parts of the body. The epiphysis or apophysis of long bones is the predominant site of chondroblastoma (CB) and accounts for 1% of all primary bone tumors [1]. The clinical manifestations encompass joint stiffness, pain, and swelling [2]. The primary treatment for CB is surgery, with the most frequently mentioned procedures being curettage with or without local adjuvants, bone grafting/cement, or cryosurgery [3,4]. The local recurrence rate (LR) ranges from 0 to 39% after treatment. Although no significant risk factors for recurrence were found, there was a higher risk associated with the location of the tumor in the epiphyseal site, being young, and having a secondary aneurysmal bone cyst (ABC) component at the time of diagnosis [5,6,7,8,9,10,11].

This study aimed to conduct a retrospective review of a cohort of 38 patients who underwent surgical treatment for CBL. We analyzed the treatment methods used, the occurrence of recurrence, any complications, and the functional outcomes. Additionally, we investigated the risk factors for local CB relapse in pediatric patients. Our findings will enhance the existing body of literature on bone tumors.

## 2. Materials and Methods

A retrospective analysis was conducted on a cohort of 38 patients diagnosed with CB who received treatment at our institution from January 2000 to December 2021. This study excluded patients who were aged 18 or older at the time of diagnosis. The surgery involved the use of different approaches for different parts of the body. A lateral approach was used for the proximal femur, a Judet approach for the scapula, a deltopectoral approach for the proximal humerus, an ilioinguinal approach for the iliac bone, and a lateral parapatellar approach for the distal femur. Additional surgical cuts and methods varied based on the location of the tumor. It can be positioned either medially or laterally. The study collected data on age, sex, filling after curettage, complications, recurrence, and functional outcomes, and all are recorded in Table 1. The medical records, radiographs, and pathology reports were also reviewed. The Enneking classification [12] was employed to assess the tumor stage; this method is widely used to assess benign tumors. The functional outcomes were assessed using the Musculoskeletal Tumor Society (MSTS) functional grading system. The assessment included evaluating pain, functional limitation, walking distance, support use, emotional acceptance, and gait using a 30-point scale. Regular clinical and radiological follow-up assessments were conducted every six months during the initial two-year period. Consequently, it was performed annually.

## 3. Statistical Analysis

Statistical analysis was conducted using SPSS software, Version 26 (IBM, Armonk, NY, USA). The mean, maximum, and minimum were used to describe continuous variables, such as age and time of follow-up. The recurrence rates were analyzed using the chi-square test with likelihood ratios.

## 4. Results

There were 22 males (57.9%) and 16 females (42.1%) with a mean age of 14.7 years (range 10–17 years), and the mean follow-up was 144.2 months (24 to 276). The most commonly reported symptom was pain (89.5%), often accompanied by localized edema and joint effusion (10.5%), resulting in functional limitations. There was no pathological fracture.

**Figure 1:**
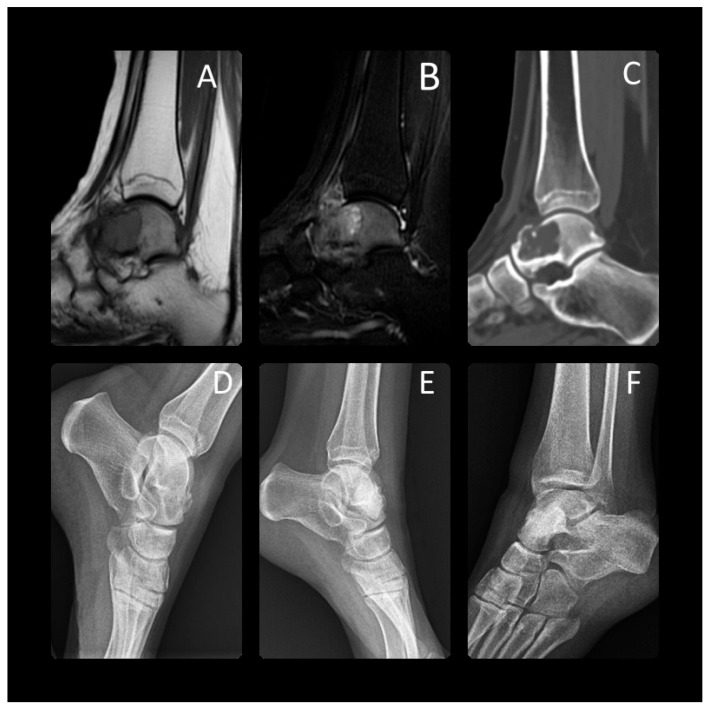
At the age of 17 years, this patient presented with chondroblastoma of the talus. A preoperative Tl-weighted (**A**) and T2-weighted (**B**) MRI, CT (**C**) and X-ray (**D**) radiograph showed a 3 cm lucent lesion within the talar neck. Expansile periosteal reaction was identified. In the distal part of the talus, particularly in the neck and the area near to the talonavicular joint, a microfracture was observed, along with considerable bone edema. This was treated with aggressive curettage and bone cementing. The final radiographs were taken 1 year (**E**) and 3 years (**F**) after surgery. Skeletal maturity shows the lesion had completely healed.

The site of CB was the proximal femur in 10 cases (26.3%) cases, the proximal tibia in 8 cases (20.8%), the humerus in 5 cases (13.2%), the distal tibia in 4 cases (10.5%), the distal femur in 3 cases (7.9%), the supracetabular region in 3 cases (7.9%), the talus in 1 case (2.6%), the calcaneus in 1 case (2.6%), the scapula in 1 case (2.6%), the lumbar spine in 1 case (2.6%), and the iliac bone in 1 case (2.6%) (Figure 1).

Intralesional curettage was used to treat these patients. The physis was open in 17 (44.7%) patients and closed in 21 patients (55.3%) at the time of surgery. The tumor was radiologically latent in 10 cases (26.3%), active in 25 cases (65.8%), and aggressive in 3 cases (7.9%), according to the Enneking staging classification [12]. Seventeen patients (44.7%) had physical involvement. One of these patients (5.9%) developed postoperative neurologic deficit and two (11.8%) underwent reoperation due to recurrence.

**Figure 2:**
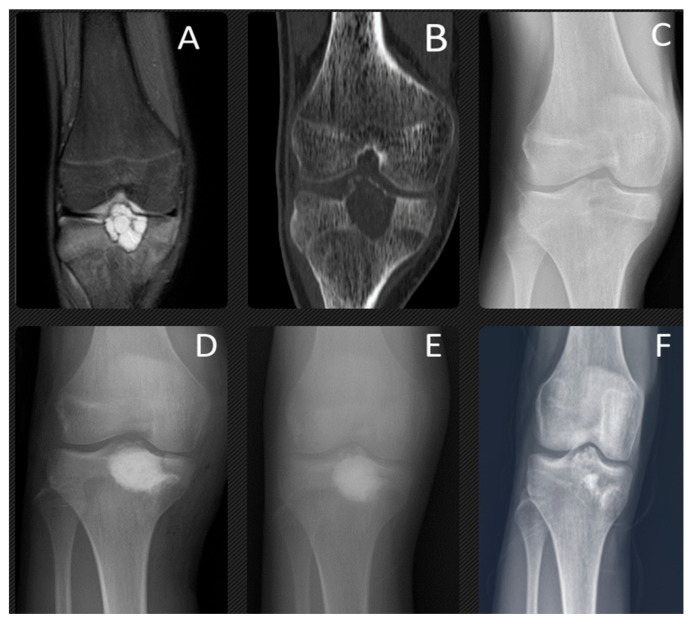
At the age of 14 years, this patient presented with chondroblastoma of the proximal tibia epiphysis, which is clearly shown on MRI (**A**), CT (**B**) and X-ray (**C**). The bone lesion was located in the proximal epiphysis of the tibia, with intralesional calcifications, an irregular sclerotic rim, and a thinned cortex. This active bone lesion crossed the physis. T2-weighted (**A**) coronal magnetic resonance images show the marrow edema of the epiphysis and metaphysis surrounding the chondroblastoma. This was treated with aggressive curettage and filled with synthetic bone graft. The final radiographs were taken 1 year (**D**), 3 years (**E**), and 7 years (**F**) after surgery. The anteroposterior (**F**) radiograph shows the lesion has completely healed and there does not appear to be any damage to the growth plate.

On histological examination, secondary aneurismal bone cysts (ABCs) were found in five cases (13.2%), including one in the proximal femur, one in the proximal tibia, one in the humerus, one in the lumbar spine, and one in the iliac bone. Twenty-one (55.3%) patients underwent trocar-needle biopsies and nine (23.7%) had frozen sections following primary surgery. The remaining eight (21.1%) patients did not have preoperative biopsies because of typical imaging findings of CB (Figure 2).

Concerning surgical treatment, curettage and filling of the bone cavity were performed in 37 patients (97.4%), and resection was performed in 1 case (2.6%). When intralesional curettage was used to treat patients, different combinations of local adjuvants were used based on the surgeon’s preference. A high-speed burr combined with phenol and cauterization was used in 22 (57.9%) patients; in 16 (42.1%) patients, only phenol and cauterization were used. Of the 22 patients who underwent intraoperative high-speed burr combined with phenol and cauterization, 2 (9.1%) experienced recurrence and 3 (13.6%) experienced complications. Of the 16 patients who underwent phenol and cauterization alone, 1 (6.3%) experienced recurrence and 2 (12.6%) experienced complications.

After curettage, the residual bone cavity was filled with cement/synthetic bone grafts in 16 (42.1%) patients, xenografts in 14 (36.8%) patients, autologous bone grafts in 3 (7.9%) patients, and allogenic bone grafts in 4 (10.5%) patients. The decision to fill the cavity with cement or bone graft was made intraoperatively after curettage. If the lesion indicated an intraosseous lesion with cortical thinning and expansile borders after the curettage, the residual bone cavity was filled with cement/synthetic bone graft. A bone graft was used when there was no cortical thinning after the curettage.

Thirty-five (92.1%) patients remained continuously local recurrence-free at a mean follow-up of 144.2 months (range 24 to 276 months), while three patients (7.9%) experienced local recurrences. A 14-year-old male patient had a recurrence of CB in the humerus seven months after curettage and local adjuvant treatment, which was successfully treated with a new aggressive curettage. Another recurrence was observed after curettage of the lumbar spine, which was treated with repeated curettage. The last recurrence was observed in the proximal tibia and was successfully treated with aggressive curettage. During the study, none of the patients had metastases.

Long-term complications occurred in five (13.2%) patients. Four (10.5%) patients experienced neuropathy after surgical treatment. Neuropraxia was found in three of the patients and peripheral neuropathy was found in one patient. This may have occurred due to stretching or compression of the nerve during surgery and spontaneously resolved in all patients during the sixth month. One (2.6%) patient experienced arthrosis, including chondropathy of the knee joint, after undergoing curettage and cementation for a tibial CB.

The MSTS score system was used to assess the functional results. The mean score was 28.3 points (17 to 30).

## 5. Discussion

Research on chondroblastoma in pediatric patients is scarce. Most chondroblastoma series exhibit a higher proportion of males. This study’s male-to-female ratio was 1.3:1, which aligns with the results of other studies [1,4,11,13,14,15]. The youngest individual was 10 years old, and the middle value of the age range was between 10 and 17 years. The majority of the patients fell within this age bracket. In our dataset, the proximal femur and proximal tibia were the most commonly occurring locations, which aligns with the previously published series [11,16]. The proximal humerus and distal femur were the most widely occurring locations in the other series, as indicated by references [1,4,13,14,17].

In 2009, Frederic Sailhan reviewed 87 cases of chondroblastoma with open physis. The average age at diagnosis and treatment was 12.5 years [15]. He observed that epiphyseal chondroblastomas exhibited a greater propensity for recurrence when compared to metaphyseal, apophyseal, and epiphyseal–metaphyseal lesions. Many authors identified an open physis as a risk factor due to the surgeon’s concern about potential damage to the growth plate. Two of the patients in our study who had recurring tumors were 13 and 11 years old. Recurrence was not linked to age or physical condition. A recent multicenter retrospective study involving 126 patients found that the average age was 18.8, which was higher than that in our study [11].

One recurrence occurred in the lumbar spine, and was associated with incomplete curettage. For chondroblastoma, surgery, specific curettage combined with bone grafting remains the cornerstone of treatment [1,16,18]. All patients in our series underwent intralesional curettage in addition to bone grafting or cementation. Treatment options included curettage combined with burr, phenol, and cauterization in twenty-two and curettage combined with phenol and cauterization in sixteen patients.

Although multiple studies [14,19] have indicated a decrease in recurrence rates with the implementation of adjuvant therapy, it remains uncertain whether adjuvant treatments cause this reduction. According to a report by Suneja et al. [1], aggressive curettage alone was generally effective in curing CB cases in a large series.

Incomplete curettage is a significant risk factor for local recurrence. Aggressive curettage is essential, despite the potential harm it can cause to the growth plate. Performing a non-aggressive curettage procedure may reduce damage to the growth plate and lead to a higher likelihood of recurrence [3]. Of the patients in our series, 17 individuals (44.7%) had physical involvement. Two individuals (11.8% of the total) required a second operation due to the reappearance of the condition. In our study, there was no significant association between the local recurrence of chondroblastoma and physical involvement (*p* = 0.443). All patients in our study underwent a rigorous procedure of removing tissue with a scraping instrument and using additional substances to enhance the treatment. Three of these patients experienced a reappearance of the condition in the same area. The combination of high-speed burr with phenol and cauterization resulted in two (9.1%) recurrences, while the group treated with phenol and cauterization alone had one (6.3%) recurrence. No correlation was observed between the type of treatment and local recurrence of CB in the pediatric population (*p* = 0.621). However, it is essential to acknowledge that the limitations of our study include the variations in treatments used over the years.

The local recurrence observed in patients with lumbar spinal chordoma is likely due to incomplete removal of the original tumor during curettage. The local recurrence rates reported in various studies ranged from 4.8% to 32%. However, our specific local recurrence rate is 7.9% [1,11,15,16]. Risk factors for local recurrence include an atypical location at coordinates [11,15], biological aggressiveness, and an aneurysmal bone cyst component at position [20].

Growth disorders have been documented in the literature as a result of aggressive curettage of chondroblastomas in patients with open physis. The documented prevalence of these growth abnormalities varies from 7% to 100% [15,21]. In a study conducted by Huang et al. [9], it was noted that 11.9% of the patients experienced premature closure of the physis due to physical injury, with five patients affected. Among these, two patients required osteotomy correction. During our study, we observed premature physis closure in four patients (10.5%) at the latest follow-up. It is noteworthy that none of the patients required corrective surgery.

Benign lung metastasis is rare, as evidenced by the limited number of documented cases in the literature, with a frequency ranging from 0.4% to 3.3% [2,3,22]. In our study and in other studies [1,10,11,15], there were no reported cases of distant metastasis during the final follow-up.

After aggressive curettage treatment was performed, several authors documented a substantial occurrence of secondary osteoarthritis. Farfalle et al. [10] conducted a study on 53 patients who received curettage as a treatment. The study revealed that the rate of local recurrence was 8% and the occurrence of osteoarthritis was 38% during an average follow-up period of 78 months (ranging from 24 to 213 months). On the other hand, Liu et al. [7] reported a single case (2.8%) of mild osteoarthritis and one instance of local recurrence in a group of thirty-six patients who underwent aggressive curettage for CB. Secondary osteoarthritis is linked to several factors, including the specific location of the lesion in the epiphyseal region, damage caused by the removal of articular cartilage, impairment of blood supply due to a direct approach, and potential tissue death caused by the use of adjuvants like liquid nitrogen or phenol. Following the curettage procedure, we assessed secondary osteoarthritis in one patient’s neighboring joints, which represented 2.6% of the total cases. According to Farfalli et al. [10], patients with talar and femoral head cartilage defects were at a higher risk of developing osteoarthritis. Factors contributing to osteoarthritis progression include the disruption of retinacular vessels in the hip and tarsal canal during surgery. Our study’s incidence of secondary osteoarthritis was lower than the findings Farfalli et al. reported [10]. This could be attributed to our study’s restricted sample size of patients.

## 6. Limitations

Our study has a few limitations. One limitation is the limited number of cases due to the rarity of CB. Additionally, the study included several anatomical locations, which could have caused variations in the results. The retrospective design is another disadvantage that made our results less conclusive.

## 7. Conclusions

Chondroblastoma, an uncommon benign tumor of cartilage origin, represents around 1% of bone tumors. Predominantly seen in young people and children, it often manifests in the proximal femur and occurs more frequently in males. The symptoms, which are generally non-specific, typically include moderate pain. Aggressive curettage is the primary treatment approach for this condition. However, this procedure can lead to complications, such as injury to the growth plate, necrosis in the epiphyseal area, and potential development of osteoarthritis. The likelihood of tumor recurrence is not directly linked to patient age, but an inadequate curettage procedure can increase the risk of tumor recurrence.

## Figures and Tables

**Table 1:** Demographic data and patient characteristics.

Characteristic	Value
Gender	22 males (57.9%), 16 females (42.1%)
Mean Age	14.7 years (Range: 10–17 years)
Mean Follow-up	144.2 months (Range: 24–276 months)
Physis Status at Surgery	Open: 17 cases (44.7%), Closed: 21 cases (55.3%)
Bone Cavity Filling	
-Cement	16 cases (42.1%)
-Xenografts	14 cases (36.8%)
-Autologous Bone Grafts	3 cases (7.9%)
-Allogenic Bone Grafts	3 cases (7.9%)
-Synthetic Bone Grafts	1 case (2.6%)
Long-term Complications	5 cases (13.2%)
-Neuropathy	4 cases (10.5%)
-Arthrosis including Chondropathy	1 case (2.6%)
MSTS Score	Mean Score: 28.3 points (Range: 17–30)

## Data Availability

The data presented in this study are available on request from the corresponding author due to privacy and ethical restrictions.
